# Kinematic Adaptations of Forward And Backward Walking on Land and in Water

**DOI:** 10.1515/hukin-2015-0104

**Published:** 2015-12-30

**Authors:** Cristina Cadenas-Sanchez, Raúl Arellano, Jos Vanrenterghem, Gracia López-Contreras

**Affiliations:** 1 Department of Physical Education and Sport. Faculty of Sport Sciences, University of Granada, Granada, Spain; 2 School of Sport and Exercise Sciences, Liverpool John Moores University, Liverpool, United Kingdom

**Keywords:** gait, kinematics, aquatic environment, land environment

## Abstract

The aim of this study was to compare sagittal plane lower limb kinematics during walking on land and submerged to the hip in water. Eight healthy adults (age 22.1 ± 1.1 years, body height 174.8 ± 7.1 cm, body mass 63.4 ± 6.2 kg) were asked to cover a distance of 10 m at comfortable speed with controlled step frequency, walking forward or backward. Sagittal plane lower limb kinematics were obtained from three dimensional video analysis to compare spatiotemporal gait parameters and joint angles at selected events using two-way repeated measures ANOVA. Key findings were a reduced walking speed, stride length, step length and a support phase in water, and step length asymmetry was higher compared to the land condition (p<0.05). At initial contact, knees and hips were more flexed during walking forward in water, whilst, ankles were more dorsiflexed during walking backward in water. At final stance, knees and ankles were more flexed during forward walking, whilst the hip was more flexed during backward walking. These results show how walking in water differs from walking on land, and provide valuable insights into the development and prescription of rehabilitation and training programs.

## Introduction

The ability to walk unaided plays a major role in people’s independence, quality of life, and participation in society (Schmid et al., 2007). It is often impaired by musculoskeletal or neurological conditions or diseases such as osteoarthritis, balance disorders, multiple sclerosis, a stroke, or cerebral palsy (Bowden et al., 2012; Patterson et al., 2012a; Routson et al., 2013). Regaining or improving the ability to walk is a primary occupation for these patients (Bohannon et al., 1988; Bowden et al., 2012; Gordon et al., 2013). Rehabilitation exercises are mainly carried out on dry-land, although, it has become common practice to use exercises with the body partly submerged in water (Prins and Cutner, 1999), typically to the level of the xiphoid process (Denning et al., 2010; Masumoto et al., 2007a).

Whilst having potential safety or motivational benefits (e.g. patients who may previously have fallen and have some fear, may well be less fearful and more motivated for rehabilitation involving walking in water), from a biomechanical point of view there are two principal reasons why walking in water may be beneficial: lowering of apparent body weight due to buoyancy force leads to reduced gravitational stresses on the musculoskeletal system, whilst the increased resistance to movement due to fluid drag forces is expected to slow down the motion and allow a patient to more consciously control their movements (Barela and Duarte, 2008; Barela et al., 2006; Orselli and Duarte, 2011). However, to our knowledge, there is a striking lack of comprehensive information about spatiotemporal and kinematic gait characteristics when walking in water. On the one hand, forward (FW) walking is one of the most common motor tasks, both for exercise programs on land and in water, as it can be practised by any age-group and with most clinical conditions (Chevutschi et al., 2009; Tsourlou et al., 2006; Volaklis et al., 2007). Backward (BW) walking, on the other hand, is also highly relevant as for some rehabilitation protocols BW walking has added benefits, for example for patients with patello-femoral pain syndrome (Chevutschi et al., 2009; Masumoto et al., 2009; Masumoto et al., 2007b), anterior cruciate ligament injuries (Chevutschi et al., 2009; Kim et al., 2010) or hamstring strains (Kachanathu et al., 2013).

Spatiotemporal gait characteristics are the most widely reported measures to identify deficiencies in walking ability on land, yet, very few authors appear to have investigated walking in water. Recently, Masumoto et al. (2009) reported that the stride frequency was higher and stride length was lower for BW walking in water, compared to FW walking in water. Also, Carneiro et al. (2012) observed that on land walking speed was lower during BW walking compared to FW walking, but in water the directional difference between the walking speeds was no longer significant. Regarding joint angles, Barela et al. (2006) did not find significant differences in the ankle, knee or hip comparing FW walking on land and in water. However, Carneiro et al. (2012) explained that BW walking in water involved more knee and hip flexion than BW walking on land or FW walking in water. Finally, regarding normal human locomotion, gait attributes of kinetic, kinematic and spatiotemporal variables are assumed to be symmetrical between lower limbs (Kodesh et al., 2012; Patterson et al., 2012b). Kodesh et al. (2012) noted that over-ground gait speed had no significant effects on leg asymmetry in healthy people, but the effect of walking in water is, to our knowledge, still unknown.

The purpose of this study was therefore to investigate spatiotemporal and kinematic characteristics under four walking conditions, combining two directions (forward and backward) and two environments (land and water).

## Material and Methods

### Participants

Eight young adults (four males, four females) volunteered to participate in the study. Their mean age, body height and mass values were 22.1 ± 1.1 years, 174.8 ± 7.1 cm and 63.4 ± 6.2 kg, respectively. Inclusion criteria were having an age between 18 and 35 years, and familiarity with a swimming pool through aquatic exercise or swimming. On the other hand, exclusion criteria were presenting a neurological or musculoskeletal disorder at the time of the study, presenting a loss of balance, or reporting pain in the lower limbs during walking.

### Measures

The dependent variables were walking speed (the average speed of the center of mass of the hip (m/s)), stride length (the distance between two consecutive heel strikes by the same leg (m)), step length (the distance between two consecutive heel strikes (m/step)), support phase duration (the total time the body is supported by one leg during one complete gait cycle (%)), step length and step time asymmetry (arbitrary units), ankle (º), knee (º) and hip (º) joints angles at initial contact (IC) and a final stance (FS) during each stride, and the medio-lateral as well as vertical displacements of the midpoint of the pelvis. Using the same method as Patterson et al. (2012a, 2012b) to calculate asymmetry, we used the left and right average values of the steps in a ratio with the largest value in the numerator so that all values for every individual were >1.0. A ratio value of 1.0 denotes perfect symmetry.

To obtain the ankle joint angles, we took the position of the heel and toe along the longitudinal axis (Z) and the position of the ankle and knee along the longitudinal axis (Z) in order to be defined in the Y-Z plane. For the knee joint angles, we took the position of the knee and iliac crest (Z axis) and the position of the ankle (Z axis) to be defined in the sagittal plane (Y-Z). The hip joint angles were defined as the line between the big toe of the foot and the hip joint centre in relation to the vertical axis (Z) through the hip joint centre defined to the Y-Z plane. Moreover, the medio-lateral and vertical displacement of the midpoint of the pelvis was calculated using the Bells’ method (Bell et al., 1990). All gait cycles were normalized in time from 0 to 100%.

### Procedures

The order of the four types of walking was counterbalanced between participants. Both on land and in water, the participants were requested to cover a distance of 10 m at comfortable speed, yet, controlled by a digital metronome (Korg TM-50) at eighty pulses per minute for land and 50 pulses per minute for water condition. To avoid the interference of the upper limbs, participants were asked to walk with arms crossed at the chest for all conditions (Carneiro et al., 2012; Grasso et al., 1998). No participants presented any impediment.

**Figure 1:**
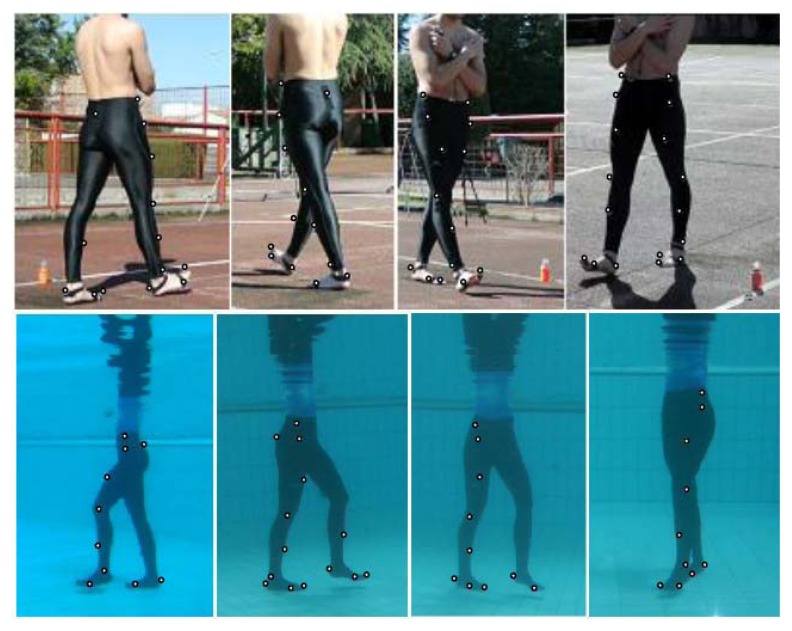
Four camera views during forward walking on land and backward walking in water, with marker positions indicated where visible to the camera

Twenty-one passive reflective markers were placed on each participant’s right and left side at the following points: a big toe, first and fifth metatarsal head, calcaneus, lateral malleolus, a mid-lateral side of the tibia, femoral epicondyle, a mid-side of the thigh, greater trochanter, sacrum and on top of the iliac crest (Figure 1).

Before any measurement, participants performed several trials to familiarize themselves with the metronome, modalities of walking, and the experimental environment. They were considered adapted when they could maintain their balance and showed coordination between the metronome pulses and their steps. The number of trials required for the familiarization was between four and six. Trials on land and in water were collected on two different days. Specifically, in water the trials were performed in a swimming pool 10 x 8 m and 120 cm deep. Such depth allowed the participants to be immersed approximately at the xiphoid process level.

The participant’s motion was recorded at 60 Hz (HD 1280x720 pixels; shutter speed 1/1000 s) with four digital cameras (1J1, Nikon VR). Video images were synchronized using an external flashing light. We recorded three cycles of the gait per modality of which the second stride (one gait cycle) was analysed. The study was approved by the Ethical Committee of the University of Granada.

### Data reduction and analysis

In order to calibrate the cameras, firstly, we digitised a reference system with a total of 66 points to provide the real dimensions of the space. Markers were manually digitized and then reconstructed to 3D coordinates using a direct linear transformation (DLT) algorithm in the land condition and a localized DLT algorithm to account for refraction in the water condition (Kwon, 1999; Kwon and Casebolt, 2006; Orselli and Duarte, 2011) using Kwon3D Software (VISOL, Inc.). To obtain an indication of reliability of outcome variables, intra-class correlation coefficients (ICC) were calculated. The intra-observer ICC ranged from 0.97 to 0.99 and inter-observer ICC ranged from 0.98 to 0.99. This demonstrated excellent reliability of the digitization process.

The raw marker coordinates were filtered using a 2^nd^ order Butterworth low-pass filter with a cut-off frequency of 6 Hz, allowing us to calculate position, velocity and angular displacement of each segment along three axes: X (medio-lateral (rightward)), Y (anteroposterior (forward)) and Z (longitudinal (upward)).

### Statistical Analysis

Means, standard deviation (SD) and confidence intervals (CI) were used to represent the studied variables. The kinematic characteristics of the two environments and directions were compared using repeated measures ANOVA. The Bonferroni correction for each category of variables was applied, resulting in alpha levels of 0.008 or 0.025. This was considered acceptable as a family-wise correction, yet, not overly conservative as if one were to correct for experiment-wide error rates (Knudson, 2009). We assumed a normal distribution of the data. All statistical analyses were performed using SPSS software version 21.0 for Windows.

## Results

### Spatiotemporal variables

Table 1 presents the mean, SD and CI of the temporal and spatial gait variables. On land, walking speed was greater than in water (F1,7=131.57; p<0.001). When participants walked FW this difference was greater than when they walked BW (F1,7=33.77; p=0.001). Walking speed was always higher when walking FW (F1,7=111.50; p<0.001), although this difference was only significant on land. Stride length was significantly higher in FW than BW walking in both environments (F1,7=128.00; p<0.001) and was greater on land than in water (F1,7=75.25; p<0.001), but showing only a significant difference when walking FW (p<0.001). Step length demonstrated the same pattern. Overall, the relative duration of the support phase was reduced in water for both walking directions with a decrease between 6 and 8% (F1,7=19.82; p=0.003). In water, higher asymmetry than on land was found when participants walked in the same direction (F1,7=40.71; p<0.001).

### Joint Angles

**Figure 2:**
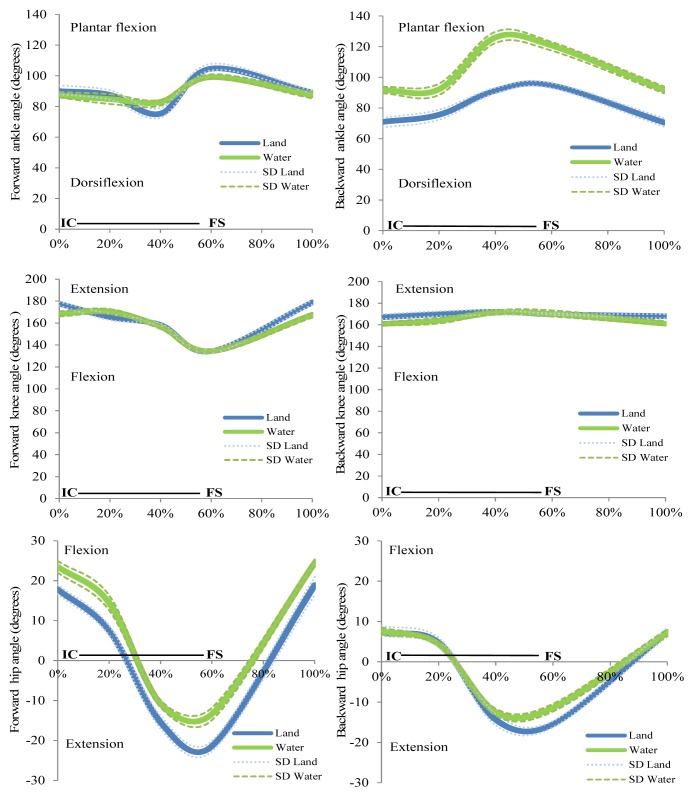
Time normalized joint angle profiles for the ankle (top), knee (middle) and hip (bottom) during walking forward (left) and backward (right) in the two environments. Dashed lines/dots represent the standard deviation (SD). Horizontal lines indicate the stance phase from initial contact (IC) to the final stance (FS). All data are normalized (n=8)

Figure 2 depicts the mean and SD of joint kinematics throughout the stride cycle. It can be seen that the ankle in FW walking was more flexed on land during the support phase (first 60% of the cycle gait approximately) and in water during the swing phase (the last 40% of stride cycle approximately). The ankle was more extended in water than on land. For the knee joint all modalities seemed to have roughly similar patterns in both conditions. In FW walking the hip angle was lower on land during the first 60% of the cycle and greater in the swing phase. In BW walking the patterns were very similar during the first 45% of the cycle, between 45 and 70% the hip angle was lower on land than in water, and for the remaining 30% of the stride cycle the hip angle increased on land compared to the water condition.

Ankle, knee and hip joint angles were compared at IC and FS in all conditions (Table 2). At initial contact (IC), the ankle angle during BW walking showed a greater dorsiflexion than during FW walking on land (p<0.001), while in water there was no significant difference. On land, the ankle during BW walking showed a greater dorsiflexion than in FW walking at the final stance (FS), whilst in water, the ankle was more dorsiflexed in FW than in BW walking at FS. For the knee angle, differences were observed between environment (F1,7=49.27, p<0.001 at IC) and direction (F1,7=28.77; p=0.001; F1,7=372.74, p<0.001 at IC and FS, respectively). At IC, the knee was more extended in FW than BW walking. In water, the knee was more flexed than on land. At FS, the knee was more extended in BW than FW walking.

The hip angle differed between environments (F1,7=47.46, p<0.001; F1,7=112.05, p<0.001) and direction (F1,7=550.10; p<0.001; F1,7=51.59, p<0.001) at IC and FS, respectively. At IC, the hip was more flexed in FW walking. Moreover, the hip angle was more flexed in water than on land, but only with a significant difference for FW walking. At FS, the hip was more flexed in water than on land, and in BW walking compared to FW walking.

### Displacement of midpoint of the pelvis

Displacement along the medio-lateral (X) axis differed according to the environment (F1,7= 267.55; p<0.001) and walking direction (F1,7=78.26; p<0.001). On land the medio-lateral displacement decreased in both walking directions compared to walking in water (p<0.001). During FW walking in water, the medio-lateral displacement was greater than BW walking. There were no significant differences between FW and BW walking on land. Vertical displacement of the midpoint of the pelvis was greater in water than on land (F1,7=76.28; p<0.001).

## Discussion

The objective of this study was to investigate kinematics of the gait cycle in healthy adults in four conditions: forward and backward walking on land and in water. We observed a number of differences between these walking conditions, which have important consequences towards the application of the various walking modalities as part of rehabilitation programmes.

Participants walked significantly slower during BW walking compared to FW walking on land, but in water this difference no longer existed. This is similar to previous findings (Carneiro et al., 2012; Chevutschi et al., 2009). Differences between environments can be explained by the fluid drag force, a lower apparent body weight and a lower comfort (due to instability) (Barela et al., 2006; Carneiro et al., 2012; Masumoto et al., 2009). In this regard, people are more careful during BW walking, where the lack of forward vision can increase problems with balance and fear of falling (Carneiro et al., 2012; Masumoto et al., 2007b). The absence of a difference between FW and BW walking in water could be due to a ceiling effect in maximal friction applied to the floor surface, which is reduced compared to walking on land due to reduced normal reaction force, combined with high hydrodynamic resistance.

On land, step and stride lengths were larger than in water which is consistent with findings in the literature (Barela and Duarte, 2008; Masumoto et al., 2012; Masumoto et al., 2008). Also, these variables were larger in FW walking than BW walking. Reduction in step length and stride length in water and in BW walking may be related to the drag force and reduced familiarity with the task (Masumoto et al., 2009). The support phase duration for walking was between 6.4 and 8.8% lower in water compared to land, which was similar to values observed in other studies (Barela and Duarte, 2008; Orselli and Duarte, 2011). Probably this change is due to the drag force of water acting on the body, increasing the swing phase duration and therefore, leaving the participants with a shorter support phase in water.

As previously reported for walking on land, the asymmetry of step length was an important measure providing information and insight into the control of walking. Our results on land confirm earlier findings that healthy adults are highly symmetrical (Kodesh et al., 2012; Lythgo et al., 2011). Despite the growing use of the aquatic environment in rehabilitation, no previous studies had analysed the asymmetries of this activity. We found that participants had more asymmetry in water compared to on land. In this context, a slight asymmetry should be considered within normal limits and may reflect exaggeration of functional differences in the contribution of each limb to propulsion and support in walking (Kodesh et al., 2012; Sadeghi et al., 2000). Additionally, the water resistance, viscosity and the water movements likely generate greater instability causing less controlled movements and increased asymmetry.

At the initial contact, the ankle was more dorsiflexed in BW walking on land than BW in water. Compared to FW walking on land, BW walking also showed a greater ankle dorsiflexion. Other authors also found differences between direction, but they did not find significant differences between environments (Carneiro et al., 2012). At the final stance, BW walking was then again associated with more plantar flexion when participants walked in water compared to walking on land. Consistent with Kodesh et al. (2012), we observed that some participants lost contact with the floor at the end of this phase because of a heel-off due to the buoyancy force in water. The increased plantar flexion is likely a compensation mechanism to try and increase contact duration with the floor.

The knee and hip were more flexed at IC in water compared to walking on land. In both environments, the hip was more flexed in FW walking than in BW walking. This is in agreement with the results of Carneiro et al. (2012), whilst in disagreement with Barela and Duarte (2008) who found the same degree of flexion in both environments. A plausible cause of these different observations might be related to the age of the participants (elderly participants) in the latter study. Taken together, these results can be explained by adaptations in water seeking to reduce the frontal area of the body segments, and therefore the fluid drag, to achieve greater mechanical efficiency in the movement. At FS, consistent to Barela and Duarte (2008) and Carneiro et al. (2012), the knee and hip were still more flexed in FW walking than in BW walking, both in water and on land.

Finally, the medio-lateral displacement of the pelvis showed an increase during walking in water, which may suggest reduced postural stability in water. We have to take into consideration, however, that for the gait without the support of arms acting as stabilizers, controlling the large hydrodynamic drag forces in water requires increased efforts and is likely the cause of these increased lateral displacements. Also in the vertical direction the displacement of the pelvis was greater in water than on land. The logical explanation lies in the action of the buoyancy force which provokes the body to be pushed upward, not only reducing the time during which the feet are in contact, but also provoking an increase of the elevation of the centre of mass. The increased vertical movements in water are likely not undesirable, as they will result in increased downward decelerations and likely greater normal reaction forces. These in turn lead to greater maximal friction forces with the floor surface, allowing for faster progression if desirable.

A limitation of our study was the small sample size to accommodate for long manual processing times of video-based digitization, so it is possible that some small, yet meaningful differences between environments or walking direction were not observed. Nevertheless, we are of the opinion that our findings represent general sex-independent modifications and that increasing sample size will unlikely change the outcome at large. Whilst both sexes were tested, comparison between males and females was beyond the scope of this study. Finally, the walking cadences in water and on land were chosen to represent what would be feasible for the majority of patients when undergoing rehabilitation, yet for the healthy individuals this may have been a slightly lower cadence than their typical walking. The reduced cadences were nevertheless expected to induce relatively minor gait adaptations compared to having to walk unnaturally fast, and that particularly in water.

In summary, the present study sheds a comprehensive light on kinematic aspects of walking in water compared to walking on land. Specifically, hydrodynamic resistance in water conditioned the stride length, whilst buoyancy led to a reduction in support phase duration and greater vertical oscillations. The hydrostatic pressure combined with the water drag induced limb movement modifications particularly concerning joint angular displacements, and somehow exaggerated asymmetry was evident in water compared to on land. These observations provide valuable anchor points for the development of rehabilitation programs in water and on land for adults. It allows the therapist to better differentiate between environment dependent adaptations and patient dependent problems with locomotion. Whilst the current study can provide a baseline understanding of adaptations to walking in an aquatic environment, there is scope for future research to advance our understanding of how gait kinematics on land and in water are further affected in specific pathological populations.

## Figures and Tables

**Table 1:** Mean, standard deviation (SD) and confidence interval (CI) of kinematic gait variables on land and in water, for different directions (forward and backward)

	Mean ± SD				95% Confidence Interval (CI)			
	
Spatiotemporal	Land		Water		Land		Water	
		
	Forward	Backward	Forward	Backward	Forward	Backward	Forward	Backward
	
Speed (m/s)	0.88 ± 0.07^a^^b^	0.58 ± 0.06^a^	0.62 ± 0.03^b^	0.55 ± 0.08	0.82–0.94	0.53–0.63	0.59–0.65	0.48–0.62
Stride length (m/cycle)	1.23 ± 0.12^a^^b^	0.90 ± 0.10^a^	0.90 ± 0.08^a^^b^	0.76 ± 0.07^a^	1.13–1.34	0.81–0.99	0.83–0.97	0.70–0.82
Step length (m/step)	0.66 ± 0.05^a^^b^	0.45 ± 0.04^a^	0.47 ± 0.04^a^^b^	0.39 ± 0.03^a^	0.61–0.70	0.41–0.49	0.43–0.51	0.36–0.42
Support phase duration (%)	66.4 ± 2.12^b^	68.8 ± 3.24^b^	60.9 ± 2.81^b^	60.0± 4.06^b^	64.6–68.1	66.1–71.5	58.5–63.2	56.6–63.4
Step length asymmetry	1.02 ± 0.02^b^	1.02 ± 0.02^b^	1.25 ± 0.17^b^	1.22 ± 0.10^b^	1.00–1.04	1.00–1.05	1.11–1.39	1.13–1.31
Step time asymmetry	1.03 ± 0.03	1.03 ± 0.03	1.11 ± 0.05	1.09 ± 0.07	1.00–1.06	1.00–1.06	1.06–1.16	1.03–1.16

a Significant differences for direction (p<0.008).

b Significant differences for environment (p<0.008)

**Table 2:** Mean, standard deviation (SD) and confidence interval (CI) of angular values at initial contact (IC) and the final stance (FS) in forward and backward directions in walking on land and in water

	Mean ± SD				95% Confidence Interval (CI)			
	
Joint angle	Land		Water		Land		Water	
	
	Forward	Backward	Forward	Backward	Forward	Backward	Forward	Backward
*At IC*								
Ankle (º)	90.0 ± 2.95^a^	71.1 ± 3.15^a^^b^	87.0 ± 3.33	91.6 ± 1.59^b^	87.6–92.5	68.5–73.8	84.3–89.8	90.2–92.9
Knee (º)	178.0 ± 1.59^a^^b^	166.1 ± 4.7^a^	168.1 ± 7.1^b^	161.2 ± 4.9	176.7–179.3	162.1–170.0	162.1–174.0	157.0–165.4
Hip (º)	17.4 ± 1.05^a^^b^	7.0 ± 1.33^a^	23.5 ± 2.02^a^^b^	7.6 ± 0.79^a^	16.5–18.3	5.9–8.1	21.8–25.2	7.0–8.3
*At FS*								
Ankle (º)	101.6 ± 6.82	95.7 ± 2.16^b^	99.1 ± 1.79^a^	119.2 ± 3.88^a^^b^	95.9–107.3	93.9–97.5	97.6–100.6	116.0–122.4
Knee (º)	135.0 ± 4.90^a^	170.2 ± 1.03^a^	131.1 ± 6.66^a^	169.0 ± 2.97^a^	130.9–139.1	169.3–171.0	125.5–136.6	166.5–171.4
Hip (º)	−21.3 ± 1.77^a^^b^	−15.2 ± 2.18^a^^b^	−13.2 ± 1.24^a^^b^	−11.3 ± 1.57^a^^b^	−22.8–−19.9	−17.0–−13.4	−14.3–−12.2	−12.6– −9.9

a Significant differences for direction (p<0.008).

b Significant differences for environment (p<0.008)

## References

[b1-jhk-49-15] Barela A, Duarte M (2008). Biomechanical characteristics of elderly individuals walking on land and in water. J Electromyogr Kinesiol.

[b2-jhk-49-15] Barela A, Stolf SF, Duarte M (2006). Biomechanical characteristics of adults walking in shallow water and on land. J Electromyogr Kinesiol.

[b3-jhk-49-15] Bell AL, Pedersen DR, Brand RA (1990). A comparison of the accuracy of several hip center location prediction methods. J Biomech.

[b4-jhk-49-15] Bohannon RW, Andrews AW, Smith MB (1988). Rehabilitation goals of patients with hemiplegia. Int J Rehabil Res.

[b5-jhk-49-15] Bowden MG, Embry AE, Perry LA, Duncan PW (2012). Rehabilitation of Walking After Stroke. Curr Treat Options Neurol.

[b6-jhk-49-15] Carneiro L, Michaelsen SM, Roesler H, Haupenthal A, Hubert M, Mallmann E (2012). Vertical reaction forces and kinematics of backward walking underwater. Gait Posture.

[b7-jhk-49-15] Chevutschi A, Alberty M, Lensel G, Pardessus V, Thevenon A (2009). Comparison of maximal and spontaneous speeds during walking on dry land and water. Gait Posture.

[b8-jhk-49-15] Denning WM, Bressel E, Dolny D (2010). Underwater treadmill exercise as a potential treatment for adults with osteoarthritis. Int J Aquatic Res Educ.

[b9-jhk-49-15] Gordon CD, Wilks R, McCaw-Binns A (2013). Effect of Aerobic Exercise (Walking) Training on Functional Status and Health-related Quality of Life in Chronic Stroke Survivors A Randomized Controlled Trial. Stroke.

[b10-jhk-49-15] Grasso R, Bianchi L, Lacquaniti F (1998). Motor patterns for human gait: Backward versus forward locomotion. J Neurophysiol.

[b11-jhk-49-15] Kachanathu SJ, Hafez AR, Zakaria AR (2013). Efficacy of backward versus forward walking on hamstring strain rehabilitation. Int J Ther Rehabil Res.

[b12-jhk-49-15] Kim YS, Park J, Shim JK (2010). Effects of Aquatic Backward Locomotion Exercise and Progressive Resistance Exercise on Lumbar Extension Strength in Patients Who Have Undergone Lumbar Diskectomy. Arch Phys Med Rehabil.

[b13-jhk-49-15] Knudson D (2009). Significant and meaningful effects in sports biomechanics research. Sports Biomech.

[b14-jhk-49-15] Kodesh E, Kafri M, Dar G, Dickstein R (2012). Walking speed, unilateral leg loading, and step symmetry in young adults. Gait Posture.

[b15-jhk-49-15] Kwon YH (1999). Object plane deformation due to refraction in two-dimensional underwater motion analysis. J Appl Biomech.

[b16-jhk-49-15] Kwon YH, Casebolt JB (2006). Effects of light refraction on the accuracy of camera calibration and reconstruction in underwater motion analysis. Sports Biomech.

[b17-jhk-49-15] Lythgo N, Wilson C, Galea M (2011). Basic gait and symmetry measures for primary school-aged children and young adults. II: Walking at slow, free and fast speed. Gait Posture.

[b18-jhk-49-15] Masumoto K, Hamada A, Tomonaga H, Kodama K, Hotta N (2012). Physiological Responses, Rating of Perceived Exertion, and Stride Characteristics During Walking on Dry Land and Walking in Water, Both With and Without a Water Current. J Sport Rehabil.

[b19-jhk-49-15] Masumoto K, Hamada A, Tomonaga HO, Kodama K, Amamoto Y, Nishizaki Y, Hotta N (2009). Physiological and perceptual responses to backward and forward treadmill walking in water. Gait Posture.

[b20-jhk-49-15] Masumoto K, Shono T, Hotta N, Fujishima K (2008). Muscle activation, cardiorespiratory response, and rating of perceived exertion in older subjects while walking in water and on dry land. J Electromyogr Kinesiol.

[b21-jhk-49-15] Masumoto K, Takasugi S, Hotta N, Fujishima K, Iwamoto Y (2007a). A comparison of muscle activity and heart rate response during backward and forward walking on an underwater treadmill. Gait Posture.

[b22-jhk-49-15] Masumoto K, Takasugi SI, Hotta N, Fujishima K, Iwamoto Y (2007b). A comparison of muscle activity and heart rate response during backward and forward walking on an underwater treadmill. Gait Posture.

[b23-jhk-49-15] Orselli MIV, Duarte M (2011). Joint forces and torques when walking in shallow water. J Biomech.

[b24-jhk-49-15] Patterson KK, Nadkarni NK, Black SE, McIlroy WE (2012a). Gait symmetry and velocity differ in their relationship to age. Gait Posture.

[b25-jhk-49-15] Patterson KK, Nadkarni NK, Black SE, McIlroy WE (2012b). Gait symmetry and velocity differ in their relationship to age. Gait Posture.

[b26-jhk-49-15] Prins J, Cutner D (1999). Aquatic therapy in the rehabilitation of athletic injuries. Clin Sports Med.

[b27-jhk-49-15] Routson RL, Clark DJ, Bowden MG, Kautz SA, Neptune RR (2013). The influence of locomotor rehabilitation on module quality and post-stroke hemiparetic walking performance. Gait Posture.

[b28-jhk-49-15] Sadeghi H, Allard P, Prince F, Labelle H (2000). Symmetry and limb dominance in able-bodied gait: a review. Gait Posture.

[b29-jhk-49-15] Schmid A, Duncan PW, Studenski S, Lai SM, Richards L, Perera S, Wu SS (2007). Improvements in speed-based gait classifications are meaningful. Stroke.

[b30-jhk-49-15] Tsourlou T, Benik A, Dipla K, Zafeiridis A, Kellis S (2006). The effects of a twenty-four-week aquatic training program on muscular strength performance in healthy elderly women. J Strength Cond Res.

[b31-jhk-49-15] Volaklis KA, Spassis AT, Tokmakidis SP (2007). Land versus water exercise in patients with coronary artery disease: effects on body composition, blood lipids, and physical fitness. Am Heart J.

